# Body composition, dietary composition, and components of metabolic syndrome in overweight and obese adults after a 12-week trial on dietary treatments focused on portion control, energy density, or glycemic index

**DOI:** 10.1186/1475-2891-11-57

**Published:** 2012-08-27

**Authors:** Kathleen J Melanson, Amber Summers, Von Nguyen, Jen Brosnahan, Joshua Lowndes, Theodore J Angelopoulos, James M Rippe

**Affiliations:** 1 Department of Nutrition & Food Sciences, University of Rhode Island, Kingston, RI, 02881, USA; 2 Department of Health Professions, University of Central Florida, Orlando, FL, 32816, USA; 3 Rippe Lifestyle Institute, Shrewsbury, MA and Celebration Health, Celebration, FL, USA

**Keywords:** Weight loss, Chronic diseases, Blood lipids, Risk factors

## Abstract

**Background:**

Given the rise in obesity and associated chronic diseases, it is critical to determine optimal weight management approaches that will also improve dietary composition and chronic disease risk factors. Few studies have examined all these weight, diet, and disease risk variables in subjects participating in recommended multi-disciplinary weight loss programs using different dietary strategies.

**Methods:**

This study compared effects of three dietary approaches to weight loss on body composition, dietary composition and risk factors for metabolic syndrome (MetS). In a 12-week trial, **s**edentary but otherwise healthy overweight and obese adults (19 M & 138 F; 38.7 ± 6.7 y; BMI 31.8 ± 2.2) who were attending weekly group sessions for weight loss followed either portion control, low energy density, or low glycemic index diet plans. At baseline and 12 weeks, measures included anthropometrics, body composition, 3-day food diaries, blood pressure, total lipid profile, HOMA, C-reactive protein, and fasting blood glucose and insulin. Data were analyzed by repeated measures analysis of variance.

**Results:**

All groups significantly reduced body weight and showed significant improvements in body composition (p < 0.001), and components of metabolic syndrome (p < 0.027 to 0.002), although HDL decreased (p < 0.001). Dietary energy, %fat and %saturated fat decreased while protein intake increased significantly (p < 0.001). There were no significant differences among the three groups in any variable related to body composition, dietary composition, or MetS components.

**Conclusion:**

Different dietary approaches based on portion control, low energy density, or low glycemic index produced similar, significant short-term improvements in body composition, diet compositin, and MetS components in overweight and obese adults undergoing weekly weight loss meetings. This may allow for flexibility in options for dietary counseling based on patient preference.

## Background

Overweight and obesity are global public health issues [1], with serious co-morbidities such as the metabolic syndrome (MetS), a cluster of risk factors associated with insulin resistance and heightened cardiovascular and diabetes risk [2]. Weight loss is a cornerstone to the prevention and treatment of metabolic syndrome [2-4]. Healthy and sustained weight loss relies on consuming a balanced, hypo caloric diet, engaging in regular physical activity, and employing cognitive skills and a supportive environment to support a healthy lifestyle [5-7]. While negative energy balance is essential for weight loss, the specifics of optimal nutritional approaches to improve body composition and reduce metabolic risks while maintaining dietary quality are still debated [8-10].

Increased serving sizes have been implicated in the development of obesity, because people tend to eat more without reporting greater satiety when they are served larger portions [11]. Laboratory studies show that increased portion sizes result in significantly greater energy intake [12], and decreased portion sizes significantly lower energy intake [13]. Thus, portion control has been advocated to reduce energy intake and manage body weight [11].

Energy density, defined as the amount of energy provided per unit weight of food, has also been implicated for body weight regulation [11]. Low energy density foods appear to affect satiety and satiation, and thus may aid in weight reduction [14]. Foods high in water and/or fibers tend to have low energy density, so they are often included in diets based on low energy density [15]. Furthermore, low energy density diets tend to be low to moderate in fat, because fat is the most energy-dense nutrient [16]. However, the satiating effects may be independent of dietary fat content [17]. When the energy density of meals or diets is covertly reduced, individuals tend to consume less, yet report greater satiety [13].

Low glycemic index (GI) diets have been advocated as having favorable affects on metabolic risk factors [18-24]. GI is defined as the incremental area under the blood glucose response curve after consumption of 50 grams of available carbohydrates from a test food, divided by the area under the curve after consumption of 50 grams of carbohydrates from a reference food (e.g. glucose or white bread) [25]. Some clinical trials have found greater weight loss with low GI diets than conventional diets [24,26], while others have not [23,27-30]. It has been proposed that low GI foods (e.g. whole grains) provide greater satiating efficiency than high GI foods (refined grains) [18,26].

Portion controlled (PC), low energy density (LED), and low glycemic index (LGI) diets have all been advocated for weight loss, but to our knowledge, no study has compared them all in subjects who are also including other components of healthy weight management, such as physical activity, cognitive skills improvement, and social support. Additionally, little work has addressed the effects of PC and LED diets on components of MetS. Thus, the aim of this trial was to compare the effects of these three different dietary approaches on body weight, components of the metabolic syndrome, and diet composition within the context of a comprehensive weight loss program. A secondary objective was to examine subjective appetite ratings in the LED and LGI groups. This study’s focus was on chronic disease prevention in overweight and obese adults who are otherwise healthy, and did not meet criteria for MetS except waist circumference.

## Methods

### Study design

A prospective, 12-week clinical intervention was implemented comparing subjects randomized to low energy density (LED) or low glycemic index (LGI) diet plans, and a similar group of subjects who had enrolled in the program’s portion-controlled (PC) plan. The trial was approved by the Florida Hospital Institutional Review Board, and all subjects read and signed informed consent forms.

### Subjects and screening

Subjects were recruited through newspaper advertisements were initially screened by telephone. Sedentary (<150 minutes of physical activity/week), weight-stable males and females, aged 25 to 50 years, with a body mass index (BMI; calculated as kg/m^2^) of 27 to 35, who were not currently taking prescription medication or over-the-counter supplements for weight loss were recruited. Exclusion criteria included diabetes, uncontrolled hypertension, orthopedic limitations, eating disorders, pregnancy or lactation, surgical medical conditions, recent weight loss, excess alcohol intake, and serious medical conditions. Eligible participants could not be currently enrolled in any commercial weight loss program (at least two weeks discontinuation required prior to the study) . During the first of two on-site qualifying visits, subjects underwent a complete physical examination, and all eligibility criteria were screened. Baseline data collected during the qualifying visits are indicated in Table 1.

**Table 1:** Baseline physical characteristics and dietary intake of individuals enrolled in a 12 week weight loss program

	LED*		LGI*		PC*		**n**
	**Mean**	**SD**	**Mean**	**SD**	**Mean**	**SD**	
Age (years)	38.8	7.0	39.1	7.1	37.9 ± 7.0		57, 59, 41
Gender	M = 7, F = 50		M = 7, F = 52		M = 5, F = 36		M = 19,F = 138
Body Mass (kg)	85.71	11.19	84.32	12.42	85.38	8.98	57, 59, 41
BMI (kg/m^2^)	31.20	2.42	31.13	2.50	31.83	2.18	57, 59, 41
Waist Circumf. (cm)	91.35	7.67	91.57	10.59	91.09	7.74	57, 59, 41
Body Fat Percentage	45.67	5.11	46.20	5.25	46.56	5.82	57, 59, 41
Systolic BP(mmHg)	114.28	11.63	113.02	10.11	112.39	8.69	57, 59, 41
Diastolic BP(mmHg)	72.21	6.97	72.42	7.13	71.12	7.27	57, 59, 41
Cholesterol (mmol/L)	5.07	0.85	5.22	1.05	5.29	1.29	57, 59, 41
Triglycerides (mmol/L)	1.32	0.70	1.53	0.91a	1.15	0.61	57, 59, 41
HDL (mmol/L)	1.37	0.26	1.42	0.33	1.44	0.31	57, 59, 41
LDL (mmol/L)	3.11	0.76	3.07	0.76	3.32	1.27	57, 59, 41
Insulin (pmol/L)	58.06	27.85	66.74	42.78	76.44	34.31	56, 59, 41
Glucose (mmol/L)	4.71	0.43	4.68	0.41a	4.93	0.61	57, 59, 41
HOMA-IR	1.78	0.92	2.03	1.38	2.18	1.24	56, 59, 41
Glc AUC (min*g/dl))	14.11	2.53	14.78	3.57	15.00	3.46	56, 58, 39
CRP (mg/L)	4.17	2.65	3.28	2.61	3.37	2.02	51, 45, 34
Energy Intake (KJ/day)	8325.74	2361.45	8578.04	2970.64	8380.13	2448.90	54, 58, 35
Energy Density (KJ/g)	4.56	1.21	4.18	1.21	4.18	1.21	55, 58, 35
Total Fiber Intake (g)	8.3	2.4^b^	8.2	2.7^b^	15.8	4.9	54, 58, 35
Energy from Fat (%)	35.7	7.8	35.8	5.7	35.4	7.3	54, 58, 35
Energy from CHO (%)	47.1	8.3	46.8	6.6	48.3	9.3	54, 58, 35
%Energy Protein	17.1	3.8	17.3	3.9	16.6	3.9	54, 58, 35
%Energy Sat.Fat	12.3	3.1	12.4	3.2	11.9	2.9	54, 58, 35

*LED, Low Energy Density; LGI, Low Glycemic Index; PC, Portion Control; BP, blood pressure; Glc, glucose; CRP, C-reactive protein; CHO, carbohydrate.

a, different from PC, p < 0.05; b, different from PC, p < 0.001.

### Intervention

All eligible subjects participated in a commercially available multi-disciplinary weight loss program (Weight Watchers) with weekly meetings to foster regular physical activity, cognitive skills, and a supportive environment. Weekly meetings lasted approximately one hour each and included weigh-ins, social support, discussions, and education. At baseline, subjects in all groups received initial individual counseling from a Registered Dietitian on how to follow their assigned dietary plan, including education materials. Recipes, shopping lists, and other guidelines specific to PC, LED, or LGI were distributed to and reviewed with the subjects accordingly. Adherence to each group’s respective diet was emphasized during weekly meetings.

Subjects in the PC dietary group were instructed on an approach that assigns point values to foods based on the energy content, dietary fiber, and total fat in defined serving sizes. Each subject was assigned an individualized target amount of point values to consume, based on current weight and a target weight loss of about 0.5-1 kilogram per week. Subjects kept track of the point values of foods consumed, to assure that their daily intake was within their points limit. In addition, guidelines regarding food choices to ensure nutritional adequacy were provided [31]. Weight loss studies using this specific approach to portion control have been published previously [30].

Subjects in the LED group were instructed to follow a plan focused on wholesome low energy density foods that have a low likelihood of being overeaten on a regular basis. Guidelines about making food choices that ensure a balanced intake were also provided [31]. In contrast to the PC group, the plan did not require eating specified amounts of a food or tracking of food choices. Rather, food intake was monitored via a periodic numeric assessment of hunger and satiety, with instructions to eat prior to getting too hungry and stopping before feeling too full [32].

Subjects in the LGI group followed a dietary plan based on foods from the Low Glycemic Index Pyramid [33]. Like the LED group but unlike the PC group, the LGI group was not prescribed specific portions of food or tracking. Instead, subjects ate ad libitum from the LGI Pyramid and followed its guidance on food choices based on GI. Subjects were encouraged to eat unrefined grains such as whole grain cereal, oatmeal, whole wheat pasta, brown rice, whole grain bread, and bulgur “in moderation.” Refined grains (white bread, white rice, grits, couscous, sweets, and potatoes) were in the “choose sparingly” section of the pyramid, due to higher GI. Guidelines to ensure nutritional adequacy were also provided to the LGI group [31]. Similar to the LED group, food intake was monitored via a periodic numeric assessment of hunger and satiety, with instructions to eat prior to getting too hungry and stopping before feeling too full.

### Measurements

Outcomes were measured at baseline and at 12 weeks, as described in the following sections.

BMI was calculated from fasting weight and height measured to the nearest 0.01 kg and 0.1 cm. Waist circumference was measured in duplicate with a flexible tape measure at the site of the iliac crest after normal expiration. Body composition was determined by validated [34] air displacement plethysmography (ADP) in a self-contained system comprised of a computer integrated dual chamber air plethysmograph equipped with a digital scale (Model 2000 A, Life Measurements, Inc, Concord, CA, USA). This methodology is sensitive to moderate changes in body composition [35]. Fasting state multiple measurements were taken with the subject in minimal, tight-fitting clothing. Percent body fat and lean body mass were calculated from body volume using the Siri equation, as with other densometric methods [34].

Blood pressure was measured by auscultation in duplicate on the non-dominant arm after the subject sat quietly for 15 minutes. A standardized two hour oral glucose tolerance test (OGTT) was performed to assess responses to a glucose challenge. Insulin sensitivity and glucose disposal were determined using the respective areas under the curve (AUC) and standardized homeostasis model assessment of insulin resistance (HOMA-IR) during the OGTT. Blood samples were obtained by venipuncture and immediately centrifuged; aliquots were frozen in dry ice and shipped to the university hospital laboratory, which is certified by the College of American Pathologists. Standardized procedures were used to analyze fasting samples for glucose, insulin, C-reactive protein, total cholesterol, LDL cholesterol, HDL cholesterol and triglycerides. Samples from after ingestion of the glucose load were additionally analyzed for plasma glucose and insulin.

### Diet composition

As part of the initial instruction, all subjects received detailed instruction on keeping research quality, 3-day food diaries using visual tools such as food models and measuring cups. Diaries were completed for two weekdays and one weekend day just prior to visits at baseline and week 12, and were reviewed in detail by Registered Dietitians at the time of receipt for clarification. Dietary intake was analyzed using the Nutrition Data System for Research Software (Nutrition Coordinating Center, University of Minnesota, Minneapolis, USA) [36], by Registered Dietitians. Nutrients of particular interest included total energy intake, total fat, saturated fat, carbohydrate, total fibers, and protein. Energy density was calculated as the total energy content of the diet (kJ) divided by the diet’s total weight (grams).

Glycemic index, weighted GI, and glycemic load (GL) were calculated for each day of self reported intake. The GL is the arithmetic product of the amount of carbohydrates consumed and the GI; it describes the overall effects of both source and quantity of carbohydrates on postprandial gylcemia [25]. The GI of individual carbohydrate-containing foods was assigned using the official website for the GI and international GI database, based in the Human Nutrition Unit, School of Molecular and Microbial Biosciences, University of Sydney [25]. If a value was not available, the GI value was imputed from a similar food or similar food combinations based on macronutrient composition. For consistency, the same GI value was assigned to a food each time it was reported from any subject. Weighted GI was calculated according to the proportion of total carbohydrate contributed by each food. Weighted GI was calculated for each food using the following formula: Σ (GI for food item x proportion of total carbohydrate contributed by item) [20]. GL was calculated using the weighted GI values for each food: (Weighted GI x grams of carbohydrate)/1000 kcal [20].

### Hunger and satiety ratings

At 6 weeks, participants in the LED and LGI groups completed a 24-hour survey to assess hunger and satiety using visual analogue scales at designated 2-hour intervals throughout the day [32]. This was done in these two groups due to aspects of their interventions associated with eating according to appetite signals. Subjective hunger and satiety were rated on a scale of 0 to 5 (0- very hungry/ravenous, 1- hungry, 2- a bit hungry, 3- satisfied/comfortable, 4- not hungry at all/full, 5- stuffed).

### Statistical analyses

Power calculations were based on body weight as main outcomes, taking into account an anticipated dropout rate of 10% in each group. Throughout the study, data were entered and stored in an Access database and in Excel spreadsheets (Microsoft 2000 for Windows®). Statistical analyses were performed using SPSS-X for Windows. These analyses consisted of descriptive measures and inferential analysis comparative measures such as paired t-tests within groups, independent t-tests between groups, and repeated measures ANOVA (for Group x Time). Statistical significance was accepted at p < 0.05 for all analyses. All data are expressed as means ± standard deviation unless otherwise specified.

## Results

### Subjects

A total of 491 individuals made phone enquiries about the study, of which 226 were deemed suitable to begin screening, 5 of whom subsequently declined participation. During in-clinic screening a further 64 were disqualified leaving 157 subjects to be randomized (57 LED, 59 LGI and 41 PC). Baseline subject characteristics, which are shown in Table 1, did not differ significantly among the three groups, except for triacylglycerols, blood glucose, and dietary fiber intake. Of the 157 adults, 87.9% were female, and on average they were classified as obese. Their blood pressure, total lipid profiles, glucose, insulin, OGTT results, and C-reactive protein values were all within normal ranges for the most part. Their reported dietary intakes were typical of the American diet. Retention rates in LED, LGI, and PC groups were 79%, 83%, and 100%, respectively. There were no significant differences in these baseline characteristics between those who did and did not complete the protocol.

### Body weight and body composition

Data on changes in body weight and body composition are shown in Table 2. Statistically relevant weight loss was achieved in each group (time p < 0.001). Likewise, improvements in body fat percentage and fat mass were also observed (time p < 0.001) while fat free mass was maintained at baseline levels (time p > 0.05). However, the changes in all variables were not different among the three groups (interaction p > 0.05).

**Table 2:** Change (week 12 baseline) in body mass and body composition after a 12 week weight loss program

	LED*		LGI*		PC*		**n**	**P**
	**Mean**	**SD**	**Mean**	**SD**	**Mean**	**SD**		
Body Mass (kg)^a^	−4.14	3.64	−3.39	2.76	−3.73	2.84	45, 49, 41	0.509
BMI ^a^	−1.36	1.34	−1.11	1.04	−1.32	1.03	45, 49, 41	0.539
Percent Body Fat ^a^	−3.87	3.34	−2.65	2.97	−2.91	2.59	36, 44, 41	0.167
Fat Mass (kg) ^a^	−4.98	3.89	−3.64	3.32	−4.00	3.2	36, 44, 41	0.219
Fat-Free Mass (kg) ^b^	0.78	4.64	−0.51	6.75	0.43	1.15	36, 44, 41	0.374

*LED, Low Energy Density, LGI, Low Glycemic Index; PC, Portion Control.

a, time p < 0.001.

b, time p > 0.05.

### Components of the metabolic syndrome

Data on the clinical measures of MetS are shown in Table 3. Waist circumference decreased with time (p < 0.001). Significant improvements were observed in various measures of glucose and insulin metabolism and inflammation, namely fasting insulin levels, glucose tolerance and insulin sensitivity (time p < 0.01) and C-reactive protein levels (time p < 0.05). An overall significant decrease in HDL (time p < 0.001) was observed. Additionally, significant reductions were seen in total cholesterol and LDL (time < 0.001). However, the changes in all variables were not different among the three groups (interaction p > 0.05).

**Table 3:** Change (week 12 – baseline) in components of the metabolic syndrome after a 12 week weight loss program

	LED*		LGI*		PC*		**N**	**p**
	**Mean**	**SD**	**Mean**	**SD**	**Mean**	**SD**		
Waist Circumference (cm)^a^	−4.06	3.65	−3.31	3.03	−2.87	3.18	36, 45, 41	0.281
Systolic BP (mmHg) ^b^	−2.44	12.46	−0.05	12.08	−3.71	13.13	36, 44, 41	0.396
Diastolic BP (mmHg) ^b^	−0.78	11.44	1.91	12.15	−2.29	9.37	36, 44, 41	0.211
Triglycerides (mmol/L) ^b^	−0.15	0.43	−0.06	0.85	−0.06	0.48	44, 49, 41	0.752
HDL (mmol/L) ^a^	−0.06	0.15	−0.11	0.20	−0.05	0.16	44, 49, 41	0.217
Glucose (mmol/L) ^b^	0.04	0.40	0.06	0.30	−0.07	0.53	43, 48, 41	0.273
Insulin (pmol/L) ^c^	−9.31	24.38	−5.35	27.22	−9.45	33.82	43, 48, 41	0.741
Glucose AUC (min*g/dl) ^c^	−1.03	2.71	−0.54	2.91	−0.69	2.23	35, 40, 32	0.715
HOMA-IR^c^	−0.30	0.82	−0.14	2.14	−0.38	1.20	42, 47, 41	0.510
C-reactive protein (mg/dl) ^d^	−0.39	2.22	−0.54	2.14	−0.52	1.51	38, 35, 32	0.938

*LED, Low Energy Density; LGI, Low Glycemic Index; PC, Portion Control; BP, blood pressure; HOMA-IR, homeostasis model assessment of insulin resistance.

a, time p < 0.001.

b, time p > 0.05.

c, time p < 0.01.

d, time <0.05.

### Diet composition

All groups significantly reduced energy intake and the percent energy consumed from fat, and saturated fat, while increasing the percent calories consumed from protein (p < 0.001). No significant differences in these dietary factors were seen among the three groups (Table 4). Average reported total fiber intakes at 12 weeks were 14.1, 11.8, and 13.4 grams in LED, LGI, and PC, respectively. As shown in Table 5, the participants in the PC group reported an intake at 12 weeks with a significantly higher GI than either the LED or the LGI group (p < 0.01). However, this difference was not observed when evaluated by either the weighted GI or GL (p > 0.05). Energy density also did not differ significantly among the three groups.

**Table 4:** Change (week 12 baseline) in dietary intake after a 12 week weight loss program

	LED*		LGI*		PC*		**N**	**p**
	**Mean**	**SD**	**Mean**	**SD**	**Mean**	**SD**		
kilojoules^a^	−2447.2	2058.5	−3270.2	2734.2	−2608.3	2032.6	33, 40, 35	0.272
% Fat^a^	−5.9	9.5	−3.2	7.9	−4.3	7.8	33, 40, 35	0.379
% Carbohydrate^b^	1.5	10.8	−0.3	8.0	1.3	9.6	33, 40, 35	0.674
% Protein^a^	5.9	7.6	4.4	5.7	3.3	5.3	33, 40, 35	0.243
% Saturated Fat^a^	−2.9	4.0	−2.5	4.3	−1.4	3.4	33, 40, 35	0.266
Fiber (g) ^a^	5.8	5.5^c^	3.6	4.5 ^c^	−2.4	5.6	33, 40, 35	<0.001

*LED, Low Energy Density; LGI, Low Glycemic Index; PC, Portion Control.

a, time p < 0.001.

b, time p > 0.05.

c, different than PC, p < 0.001.

**Table 5:** Glycemic and Energy Density values of the three study diets as reported after 12 weeks

	LED*		LGI*		PC*		**N**	**p**
	**Mean**	**SD**	**Mean**	**SD**	**Mean**	**SD**		
Glycemic Index	40.15	8.64 ^a^	42.43	7.35 ^a^	46.69	7.74	33, 41, 41	0.002
Weighted GI	30.04	6.50	30.71	5.81	31.59	6.25	33, 41, 41	0.556
Glycemic Load	54.39	30.14	44.75	27.86	41.58	21.32	33, 41, 41	0.108
Energy Density (kJ/g)	3.68	1.38	3.89	1.38	4.02	1.05	33, 41, 41	0.512

*LED, Low Energy Density; LGI, Low Glycemic Index; PC, Portion Control; GI, glycemic index.

a, different than PC.

### Hunger/satiety ratings

Data on hunger and satiety ratings for the LED and LGI groups are shown in Table 6. Twenty-five questionnaires were available for analysis at Week 6. At least one participant from each group responded at a total of eight time points. To compare between-group differences, the mean rating of each group for each time point was determined. No differences in hunger/satiety rating were found between the two diets at any time point (p > 0.05).

**Table 6:** Hunger/satiety rating group means by time at week 6 in two dietary treatment groups who focused on internal appetite regulation while enrolled in a 12-week commercial weight loss program

**Time**	**LED**		**LGI**		**p**
	**Mean**	**SD**	**Mean**	**SD**	
0800	2.53	0.833	2.71	1.11	.724
1000	2.33	1.11	2.60	0.699	.754
1200	1.93	1.93	2.00	2.0	.896
1400	3.00	0.845	2.60	1.17	.212
1600	2.35	0.841	1.70	1.41	.313
1800	2.26	2.26	2.60	2.60	.546
2000	2.93	0.883	3.20	0.918	.775
2200	2.33	2.33	2.20	2.20	.167

*LED, Low Energy Density; LGI, Low Glycemic Index.

## Discussion

This 12 week clinical trial found comparable benefits from three dietary approaches to weight loss on outcomes related to body weight and composition, components of MetS, and dietary composition in overweight and obese sedentary adults. . Diet composition changed significantly in similar ways among the three groups. Of interest, the weighted GI, GL, or energy density were not significantly different among the groups. Thus, instruction on these three dietary approaches resulted in different means to similar ends.

In assessing weight loss diets, considerations must be given to changes in body composition, chronic disease risk factors, diet composition, and appetite [8]. Additionally, individuals must determine what will suit their lifestyle and personal preferences so that they can sustain the diet for long periods [8]. Results of this trial indicate that over 12 weeks, PC, LED, and LGI approaches produce analogous changes in measured outcomes within a comprehensive weight loss program.

Weight loss was achieved using PC, LED, and LGI. These findings support those of some previous studies, which also found that low GI dietary plans do not result in weight reduction beyond that of traditional dietary plans [23,27-30]. In a similar 12-week study with 129 women on four diets differing in GI and protein, weight loss did not differ among the groups, and was comparable to the current study [24]. A study that compared a reduced GL diet to a portion-controlled plan showed significantly greater weight loss at 12 weeks in the reduced GL group, but these between-group differences were not sustained thereafter [26]. Other studies have demonstrated better body composition improvements on low GI or low GL diets compared to other approaches [24,37]. Lack of consistency among studies might be related to the actual GL of the diets consumed, and/or to the additional components of the weight loss program in the current study.

Both weight loss [3,4] and lowered dietary GL [38] have been associated with reduced MetS risk factors. The overweight and obese subjects in this study were not clinically hyperglycemic or hyperinsulinemic, but improvements were seen in all three groups. Furthermore, C-reactive protein, a marker of inflammation and cardiovascular disease risk [38], was lowered regardless of dietary approach. Even modest weight reduction can profoundly reduce MetS risk [3], as shown in this study, and it was achieved in three different ways. All three interventions produced a similarly reduced dietary GL, which might help explain the similar reductions in risk factors. Total and LDL cholesterol did not decrease significantly in this trial, which might be due to the fact that the subjects were not hypercholesterolemic at the outset. HDL cholesterol decreased slightly in all groups, which is sometimes seen in studies that replace much of the dietary fat with carbohydrate, particularly in insulin-resistant individuals [39,40]. However, at week 12, the LED, LGI, and PC groups consumed dietary carbohydrate at 49%, 46%, and 49% of energy intake, which is within the 45-60% range recommended for diets to reduce MetS risk [3,4]. Weight loss studies with reduced GL diets similar to the present study have observed increased HDL cholesterol over longer time periods [26], so it is possible that the trend seen here in HDL cholesterol would have become more favorable with time.

The dietary plans in this trial were followed within the context of a comprehensive weight-loss program. Thus, the results cannot be ascribed solely to the diets alone [41,42]. All groups attended weekly meetings of similar structure, which should minimize independent effects of intervention sessions. All aspects of the protocol were identical except for the dietary approaches.

After 12 weeks, the PC group reported a significantly higher GI than the other groups, but weighted GI or GL did not differ. The PC intervention emphasized portions more than food types, so participants likely incorporated foods with higher GI values [30]. Conversely, both the LGI and LED plans emphasized foods that are rich in fiber and low in added sugars and refined starches, because these tend to be low in GI and low in ED. GL is calculated using both the quantity and quality of foods , which may help explain the similar GL among the three groups [25,37]. The PC group focused on decreasing overall food quantity, so that would help reduce the GL. Instructions for the LED group did not address GI at all, but some overlap may exist in the food choices, particularly due to fiber-rich foods having low ED. At 12 weeks, reductions of fiber intake in the PC group are likely due to high values reported at baseline, followed by reduced total food intake over the intervention. At baseline, the LED and LGI groups reported lower fiber intakes, which increased over the intervention due to the instructions related to high fiber foods. Similar fiber intakes might have contributed, at least in part, to the similar energy density of the diets among the three groups.

LGI and LED diets have been promoted for weight loss due to effects on increased satiety [30,33]. A previous 12-week study examined a dietary plan similar to the PC group in the current study, with and without reductions in GI [30]. Although weight loss did not differ according to the GI manipulation, the lower GI version of the diet was associated with reduced hunger and heightened satiety ratings. In the current study, similar 6-week hunger and satiety ratings between the LGI and LED groups suggest that these diet plans did not produce different satiety effects.. Similar appetite ratings may be attributed to the comparable calculated GL, ED, and fiber intakes between the two dietary plans. Both groups received the same counseling on recognizing and responding to internal cues and hunger signals [32].

This study only examined a 12-week period, so longer-term studies are needed to ascertain whether the results would continue to follow the same trends. Unequal gender distribution among the subjects also indicates that future studies should include recruitment efforts targeted more towards males. Another limitation of the current study is the dropout rate, although the rates are lower than many other studies, and dropouts are common, due to challenges of following prescriptive diets over long periods, and the demands of subjects’ time for laboratory visits [20,27]. Power calculations had taken anticipated dropout rates into account. As with most free-living human nutrition research, food intake was self-reported. However, the registered dietitians instructed subjects carefully on accurate dietary recording, and reviewed the 3-day food diaries in detail with subjects. Additionally, only the LED and LGI groups were randomized, and the PC group served as an extra comparison group who enrolled in the program. This may explain their full completion of the study. However, their data provide insight into results that may occur in the ‘real world’ situation of people choosing such a program. It is interesting to note that their dietary and other changes were similar to the other two groups.

Strengths of this study include the similar multi-disciplinary approach taken in all groups, including diet, physical activity, behavior modification, and regular social support [6,7]. These, which follow national and international guidelines [1-3,31], produced recommended rates of weight loss of almost 5% [3], which have been associated with significant reductions in risk for several chronic diseases [1,4]. Further, multiple health-related outcomes were measured.

## Conclusions

The major finding of this 12-week clinical intervention trial is that three different dietary approaches to weight loss produced similar improvements in body composition, metabolic parameters, and diet composition. Thus, individuals attempting healthy weight loss should have flexibility in choosing the plan that fits their personal preferences.

## Competing interests

JMR has received consulting fees and research grants from Weight Watchers International. The remaining authors declare that they have no competing interests.

## Authors’ contributions

KJM assisted with dietary and appetite aspects of the protocol, data interpretation, and wrote the final manuscript. AS, JB, and JL performed subject instructions, data collection and data entry. AS drafted the initial manuscript. TJA performed statistical analyses. JMR and TJA contributed to the design of the study and helped with the editing of the paper. All authors read and approved the final manuscript.
